# A Challenging Case of Generalised Anxiety Disorder and Recurrent Depressive Disorder, Unspecified

**DOI:** 10.1192/bjo.2024.678

**Published:** 2024-08-01

**Authors:** Eileen Moss

**Affiliations:** Holywell Hospital, Antrim, United Kingdom

## Abstract

**Aims:**

This case presentation is on a 54 year old female patient. Prior to 2023, she had never had an inpatient admission for her mental health. She was referred to her local community mental health team in May 2023 as they were concerned that she was suffering from panic disorder. The GP referral stated that this lady was suffering from anxiety and panic attacks. At the time of the GP referral, she was on maximum doses of escitalopram, propranolol and zolpidem (and she had been on these maximum doses for three years prior to the referral). This lady has significant caring responsibilities (she has a brother who is severely disabled and she lives with him and she is his main carer). She sustained an injury to the tip of her left index finger in April 2023 and this injury seemed to cause an acute deterioration in her mental health.

**Methods:**

This lady had her first inpatient admission in June 2023 and at that time she was treated for the following: mixed anxiety and depressive disorder. She was discharged to the care of her Community Mental Health Team at that time. Post-discharge, her mental state started to deteriorate and she waded into a river and she also made a serious hanging attempt. After this hanging attempt, she sustained multiple rib fractures, a pleural effusion and atelectasis. She also ended up in ICU following this suicide attempt. She was re-admitted to Holywell Hospital in November 2023.

**Results:**

During her second inpatient admission, it became clear that this lady is very medication-seeking. She was treated for the following mental health conditions on her second inpatient admission in 2023: Generalised Anxiety Disorder and Recurrent Depressive Disorder, Unspecified. Help was also sought from an Addictions Specialist on the second inpatient admission. This lady was given five ECT sessions on her second inpatient admission but it was felt that this was making her more agitated so it was stopped.

**Conclusion:**

This patient is currently still an inpatient and she is hoping to attend a specialist addictions unit when she is discharged from hospital. I will follow her progress with interest. I found this case to be an interesting one as it forced me to consider how to best manage a patient who is very medication-seeking.

